# Physician’s prescription pattern in young infants with upper respiratory infections/cough and cold in emergency department

**DOI:** 10.12669/pjms.36.2.1240

**Published:** 2020

**Authors:** Caner Turan, Ali Yurtseven, Eylem Ulas Saz

**Affiliations:** 1 Dr. Caner Turan, MD. Department of Pediatrics, Division of Emergency Medicine, Ege University School of Medicine, Izmir, Turkey; 2 Dr. Ali Yurtseven, MD. Department of Pediatrics, Division of Emergency Medicine, Ege University School of Medicine, Izmir, Turkey; 3 Prof. Dr. Eylem Ulas Saz, MD. Department of Pediatrics, Division of Emergency Medicine, Ege University School of Medicine, Izmir, Turkey

**Keywords:** OTC, Cold, Cough, Decongestant, Prescription, Child

## Abstract

**Objective:**

Despite the national/international warnings and little evidence as to whether over-the-counter cough and cold medications (OTC-CCM) are effective, physicians frequently overprescribe, parents overuse these drugs and antibiotics for URTIs in young child. This study aimed to determine the prescription pattern of over-the-counter cough and cold medications (OTC-CCM) in children less than two years.

**Methods:**

This was a cross-sectional study in which we collected physicians prescriptions in young infants less than two years of age with ARI (acute respiratory infections) who visited pediatric emergency department (ED) between September 2017-April 2018 and received prescription with OTC-CCM enrolled. Infants who did not receive OTC-CCM were excluded.

**Results:**

During the study period 2476 infants presented to the ED and 1452 (58.6%) had prescription with OTC-CCM. Analyzing the prescription details revealed that 63.8% was with decongestants, 53.5% antitussive and 52.7% antibiotics. One third of the prescriptions with these medications were written by pediatricians (p=0.001). Physicians had tendency to prescribe antibiotic if the infants had fever higher than 38°C (p=0.02).

**Conclusion:**

We observed that international and national warnings did not show a considerable impact on the prescription pattern. Despite international and national reports, physicians frequently prescribe OTC-CCM in infants.

## INTRODUCTION

Acute cough due to upper respiratory tract infection (URTI) is one of the most common symptoms in children. Although most URTI and colds in children under two years of age don’t have serious complications, they can cause worry to caregivers, and are among the top reasons for visiting emergency departments (EDs).^1^,^2^ Over-the-counter cough and cold medications (OTC-CCM) is frequently preferred as a first-line treatment in case of URTI, but there is little evidence as to whether these drugs are effective.^3^ The OTC-CCM preparations are usually a combination of at least two types of medications such as antihistaminics, antitussives, expectorants, decongestants, and antipyretics.^2^,^4^ Although life-threatening and lethal adverse effects have been reported (arrhythmias, hallucinations, death and encephalopathy-central nervous system depression) with use of OTC-CCM, these are still continue to be prescribed by physicians and used by parents/caregivers.^5^,^6^ FDA recommends that these medications should not be used for infants and children under two years of age.^7^ Similar national bulletin outlining of avoiding use these medicines in children under six years of age.^8^,^9^

In 2015, Republic of Turkey Ministry of Health issued guidelines discouraging use of these medications in children under six and abolished the re-payment these medicines in children under two. This study aimed to evaluate and document the current burden of OTC-CCM prescribed by physicians for children younger than 2. It also examined some prescriptions details such as patients /physicians demographics, physician specialty, and clinical characteristics and drugs ingredients.

## METHODS

Children who were younger than two years of age presented to ED with URTI symptoms were included. The study period was from September 2017 to April 2018. The recruitment site was Children’s Hospital of Ege University Emergency Department (ED). The prescriptions written by the first physicians were also investigated.

This was s a questionnaire based cross-sectional study was including 15 questions. Prescription details, patients’ demographics, first physician’s characteristics (who prescribed) such as specialty and indications for prescription also recorded. All patient’s follow-up on the ED also reviewed. General practitioner is only graduated from a medical school in Turkey; however, family care physician is trained at least three years postgraduation (in internal medicine, pediatrics, surgery). Parents were recruited face-to-face by one of the authors. Approval for the study was taken from the Medical School Clinical Trials Ethical Committee (Ref. No. 17-1.1/1, dated July 3, 2017).

### Classification of over counter medicines

Anatomic Therapeutic Chemical (ATC) classification system was used by World Health Organization (WHO) to classify this medicine.^10^ The OTC-CCM preparations are usually a combination of following medications such as antipyretics (paracetamol, ibuprofen, acetylsalicylic acid), antihistaminics (chlorpheniramine, promethazine, oksolamin, triprolidin, doksilamin), ethanol (menthol), antitussives (codeine, dionin, butamirat, levodropropizin, oksolamin), expectorant (guaifenesin, potassium iodide, glyceryl guaiacolat) and decongestants (phenylephrine, pseudoephedrine, ephedrine).

### Statistical Analysis

Package for the Social Sciences 22.0 package program (SPSS Inc; Chicago, IL, USA) was used for the analysis of data. Continual values of descriptive statistics were given as average mean ± standard deviation (SD). Frequency analysis indicating incidence /frequency was stated in number and percentage (%). Mann Whitney U test was used in the comparison of averages that do not match with normal distribution. Correlation analyses were conducted by using Spearman correlation analysis. Statistical significance was accepted to be p<0.05.

## RESULTS

**Fig.1:**
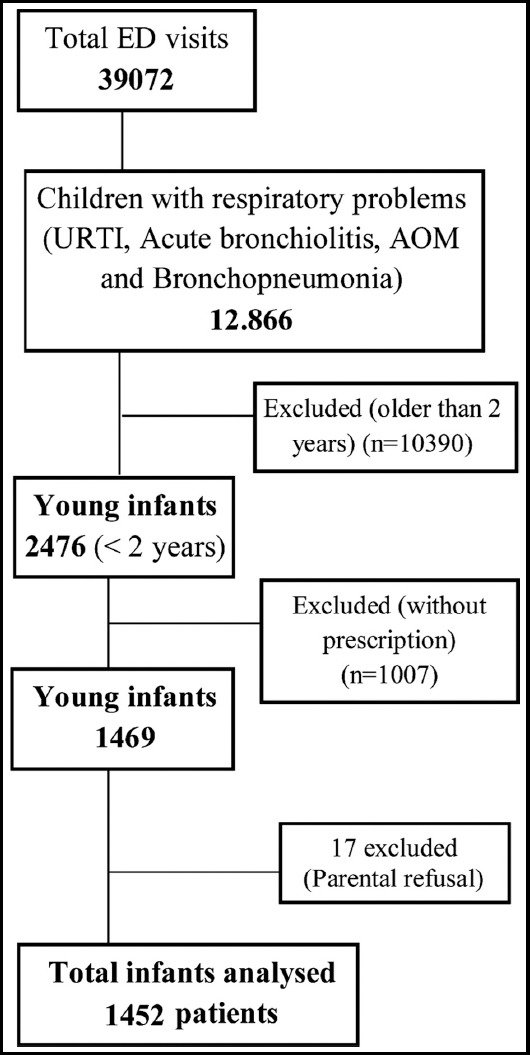
Flow chart of study enrollment.

A total of 39.072 patients presented to our ED during the study period. The rate of URTI was 32.9% (n=12866), 2476 of them were younger two years of age, only 1469 patients had a prescription. Since 17 parents refused to provide information, the final analysis was performed for 1452 patients (Fig.1). Male/female ratio was 0.8; the mean age of children was 12.3 months (SD ±6.1; min 15 days – max 24 months). The distribution of first physicians who made the initial examination and wrote the prescription was family care physicians (44.7%), pediatrician (28.9%), pediatric resident (13.3%) and practitioner (13%). Patients applied to the tertiary ED because their complaints continued and they were referred by physicians. The mean time for ED presentation following the initial examination (by former physician) in these children was 2.3 days (SD ± 0.95, min 1-max 4 days) (Table-I).

*URTI: Upper Respiratory Tract Infection,

AOM: Acute otitis Media.

**Table-I:** The patient characteristics, clinical features, diagnosis for OTC-CCM prescription and physician specialty.

***Patient characteristics***	
***Male/female ratio***	0.8
***Age*** (months) (mean, ±SD)	12.3 (±6.1)
***Admission time*** (day) * (mean, ±SD)	2.3 (±0.95)
***Complaints [n (%)]***	
Cough	1204 (83)
Nasal flow/congestion	915 (63)
Fever	795 (54.8)
Wheezing	203 (14)
Otalgia	43 (3)
Abdominal pain	28 (1.9)
Headache	27 (1.8)
***Diagnosis [n (%)]***	
URTI	1082 (74.5)
Acute bronchiolitis	158 (10.9)
Acute otitis media	136 (9.4) 74 (5.1)
Bronchopneumonia	
***Types of OTC-CCM prescription [n (%)]***	
Oral decongestants	926 (63.8)
Antitussive	777 (53.5)
Antibiotics	765 (52.7)
Nasal decongestants	41 (2.8)

OTC-CCM: over-the-counter cough and cold medications, SD: standard deviation, URTI: upper respiratory tract infection.

Physicians preferred prescribing OTC-CCM mainly for cough (n=1204; 82.9%), congestion (n=915; 63%) and fever (n=795; 54.8%) (Table-I). The most common diagnosis in the prescription of these medications was respectively; URTI (74.5%), acute bronchiolitis (10.9%), acute otitis media (9.4%) and bronchopneumonia (5.1%). Prescription details revealed that most physicians were more likely to recommend oral decongestants (63.8%), antitussive (53.3%), antibiotics (52.7%) and nasal decongestants (2.8%) (Table-I). The frequency of prescribing antibiotic among all physicians was 52.7%. Although they were more likely to recommend for infants less than 12 months (48.1%), no statistically difference was found between the age and antibiotic prescription rate. Physicians had tendency to prescribe antibiotic if the infants had fever higher than 38 °C (p=0.029).

OTC-CCM prescription rate in the whole study group was 67.4%. Prescription rate was statistically different among physician’s specialty with predominance of family care physicians (51.4%). On the other hand, pediatric residents and general practitioner prescription rate were similar with 7.9 and 10.8% respectively. One third of the prescriptions with these medications were written by pediatricians (p=0.001) (Table-II). The subgroups analysis (ingredients of these medications) revealed that the lowest prescription rate of decongestant and antitussive medicines was found in pediatric residents and general practitioner (p=0.016, p=0.007, respectively) (Table-III). The distribution of discharge diagnosis in the study group (ED) were; URTI (48.8%), acute bronchiolitis (24.8%), acute otitis media (10.3%), bronchopneumonia (9.4%), acute gastroenteritis (4.5%) and sepsis (2.1%).

**Table-II:** Comparison of physician specialty with OTC-CCM Drugs Prescription.

OTC-CCM Drugs			*p	

	Total	Prescribed [n (%)]	Unprescribed [n (%)]
Physician specialty	1452	980	472	0.001
Family Care Physicians	650	504 (51.4)	146 (30.9)	
Pediatrician	419	292 (29.8)	127 (26.9)	
Pediatric Resident	193	78 (7.9)	115 (24.3)	
General Practitioner	190	106 (10.8)	84 (17.8)	

OTC-CCM: over-the-counter cough and cold medications.

**Table-III:** Comparison of physician specialty who prescribed oral decongestants and antitussives.

Physician specialty					*p

	Family Care Physicians [n (%)]	Pediatrician [n (%)]	Pediatric Resident [n (%)]	General Practitioner [n (%)]	
***Oral Decongestants***					
Prescribed	460 (49.7)	282 (30.5)	72 (7.7)	112 (12)	0.016
Unprescribed	190 (36.1)	137 (26)	121 (23.1)	78 (14.8)
***Oral Antitussives***					
Prescribed	401 (51.6)	177 (22.8)	50 (6.4)	149 (19.2)	0.007
Unprescribed	249 (36.9)	242 (35.9)	143 (21.2)	41 (6)
***Antibiotics***					
Prescribed	312 (40.8)	253 (33.1)	98 (12.8)	102 (13.3)	>0.05
Unprescribed	338 (49.2)	166 (24.2)	95 (13.8)	88 (12.8)
***Nasal decongestants***					
Prescribed	6 (14.6)	17 (41.5)	8 (19.5)	10 (24.4)	>0.05
Unprescribed	644 (45.6)	402 (28.5)	185 (13.1)	180 (12.8)	

## DISCUSSION

OTC-CCM use in children is still high despite the national or international warnings against their use, doubtful efficacy and documented toxicity. No scientific data exist supporting the usage of medicines for cold and cough in the pediatric population.^2^,^11^-^13^ Despite this fact (national and international warnings), negative reliability reports and low activity profile, previous studies showed that physicians keep prescribing these medications.^14^-^16^ To the best of our knowledge, this is the first report from Turkey that has examined the prescription rate of these medications in children less than two years of age.

Concerns about the safety and efficacy of these medications have led to a recent US Food and Drug Administration public health advisory against their use in children < 2 years. The FDA warning was followed by other countries and finally Turkey did it at 2015. A recent study from Europe has shown that these medications use remains high despite the national warnings against their use. Overall prescription rates for OTC-CCMs increased in the Netherlands, where no national warning was issued, however a significant reduction was seen in Italy where a specific warning rules existed.^14^

Although, the prescription rates for OTC-CCMs decreased after the national warnings in Europe, we observed that our national warnings did not show a considerable impact on the prescription pattern.^14^ Furthermore, in our cohort family care physicians and pediatricians tend to prescribe more OTC-CCM and mostly for cough. Since we were investigating only the physician specialties and prescription rate this study does not explain the causes of prescriptions.

In our study most physicians use these medications for respiratory tract infections and otitis media. Nevertheless, many randomized trials, systematic reviews, and meta-analyses demonstrated that they have not been proven to work any better than placebo in children and may have serious adverse effects.^11^,^12^,^17^-^19^ These medications have been associated with life-threatening toxicity especially in children younger than two.^19^-^22^ That’s why our study especially focused on this age group who were taking medications upon presentation. Since the metabolism, clearance, and drug effects may vary according to age, young children at greatest risk for enhanced toxicity. Although, previous study has also showed that the use of decongestants does not change the natural course of the disease in the treatment of acute otitis media^23^, in our group physicians were prescribing local or systemic decongestants or antihistamines for otitis media. The dosages at which OTC-CCM can cause toxicity or death in children aged <2 years are not known. Even FDA does not have approved dosing recommendations for clinicians prescribing these medications for this age group.^13^,^16^ Prescribing these medicines seemed to be associated with hospital, and physician specialty. We believe that more strict warning rules required especially for pharmacies and parents to use of cough medications in this age group. Physicians should prescribe these medications with caution when treating children aged < 2 years. In children with URTI, antibiotics have little significant impact on either the course of illness or the likelihood of suffering complications. Majority of ambulatory antibiotic prescriptions were for the treatment of otitis media, sinusitis, pharyngitis, bronchitis, and URTIs in the US.l,^24^ Our study showed that physicians prescribed antibiotic for similar specific acute respiratory infections. Although, in this study authors have focused on use of them, the high frequency of prescribing antibiotics was also noticed. Our group physicians frequently prescribed antibiotics for URTIs/other infections when they believe infants deserve it (less than 12 months and fever >38°C). According to recent estimates, more than a quarter and up to one half of all antibiotics prescribed in U.S. ambulatory care are inappropriate.^24^,^25^ Turkey is the most prescribing antibiotics country with the highest resistance in OECD (The Organization for Economic cooperation and Development) countries.^26^ One explanation for the excessive use of antibiotics in our country is the medical malpractice system. Physicians in Turkey are at high risk of violence and face considerable legal pressure. To protect themselves against legal proceedings and violence physicians have been found to resort to defensive medicine. Overprescribing antibiotics may constitute a form of defensive medicine: physicians may feel inclined to prescribe an antibiotic against their own clinical judgment because they believe that the antibiotics present a safeguard against serious bacterial infections, which may trigger a malpractice claim if left untreated.

### Limitations of the study

Study design and time period of study are major limitations. Since fall and winter are the peak times for many viral illnesses and URTIs the study period picked to cover this season. Also, it is a single center study and our results demonstrate a regional data and cannot be reflected of all national perspective.

## CONCLUSIONS

Despite international and national warning reports physicians frequently overprescribe, parents overuse OTC-CCM and antibiotics for URTIs in young children. Family care physicians and pediatricians tend to prescribe more OTC-CCM and mostly for cough. Also, physicians frequently prescribed antibiotics for URTIs/other infections when they believe infants deserve it (less than 12 months and fever >38 °C). Due to the risks for toxicity, absence of dosing recommendations, and limited data of effectiveness in this age group, physicians should not prescribe and parents should not administer these medications. Public education, refresher training program for health personnel, national reports or feedback to physicians who keep prescribing may be effective to reduce unnecessary prescribing of antibiotics/cough medicines.

### Authors’ Contribution:

***CT, AY, EUS:*** Surgical and Medical Practices.

***CT, EUS:*** Concept.

***EUS, CT:*** Design. Data Collection, Literature Search and Writing

***CT:*** Analysis or Interpretation..
